# Prevalence of Metabolic Syndrome One Year after Delivery in Finnish Women at Increased Risk for Gestational Diabetes Mellitus during Pregnancy

**DOI:** 10.1155/2013/139049

**Published:** 2013-03-20

**Authors:** Jatta Puhkala, Tarja I. Kinnunen, Tommi Vasankari, Katriina Kukkonen-Harjula, Jani Raitanen, Riitta Luoto

**Affiliations:** ^1^UKK Institute for Health Promotion Research, P.O. Box 30, FI-33501 Tampere, Finland; ^2^School of Health Sciences, University of Tampere, FI-33014 University of Tampere, Finland; ^3^National Institute for Health and Welfare, P.O. Box 30, FI-00271 Helsinki, Finland

## Abstract

*Background*. Women with a history of gestational diabetes mellitus (GDM) are at increased risk for metabolic syndrome (MeS) after delivery. We studied the prevalence of MeS at one year postpartum among Finnish women who in early pregnancy were at increased risk of developing GDM. *Methods*. This follow-up study is a part of a GDM prevention trial. At one year postpartum, 150 women (mean age 33.1 years, BMI 27.2 kg/m^2^) were evaluated for MeS. *Results*. The prevalence of MeS was 18% according tothe International Diabetes Federation (IDF) criteria and 16% according toNational Cholestrol Education Program (NCEP) criteria. Of MeS components, 74% of participants had an increased waist circumference (≥80 cm). Twenty-seven percent had elevated fasting plasma glucose (≥5.6 mmol/L), and 29% had reduced HDL cholesterol (≤1.3 mmol/L). The odds ratio for the occurrence of MeS at one year postpartum was 3.0 (95% CI 1.0–9.2) in those who were overweight before pregnancy compared to normal weight women. *Conclusions*. Nearly one-fifth of the women with an increased risk of GDM in early pregnancy fulfilled the criteria of MeS at one year postpartum. The most important factor associated with MeS was prepregnancy overweight. Weight management before and during pregnancy is important for preventing MeS after delivery.

## 1. Introduction 

Metabolic syndrome (MeS) is defined as a cluster of atherosclerotic risk factors, including abdominal obesity, elevated serum triglycerides, decreased HDL cholesterol, elevated blood pressure, and elevated serum plasma glucose [1–3]. Insulin resistance is a central feature in the pathogenesis of MeS [4] in addition to an unhealthy diet and physical inactivity promoting overweight and genetic factors [1, 5–7]. As obesity increases worldwide, this leads to an increased incidence and an earlier onset of MeS [3, 8, 9].

Gestational diabetes mellitus (GDM), a disorder in glucose and insulin metabolism, is one of the most common complications in pregnancy [10]. Depending on the population and the diagnostic criteria used, the prevalence is roughly 1%–14% of pregnancies [10, 11]; and the occurrence is increasing worldwide [12, 13]. The most important risk factors for GDM are prepregnancy overweight, high maternal age and a family history of type 2 diabetes [14]. Women with a history of GDM are at increased risk of developing type 2 diabetes and also MeS after delivery [15–17]. Among Canadian women with a history of GDM, the prevalence of MeS was 20% at as early as three months postpartum [18]. According to studies from the USA and Denmark, approximately 30%–40% of women with a history of GDM develop MeS by ten years postpartum [19, 20]. 

The aim of this study was to determine the prevalence of MeS and its components at one year postpartum among Finnish women who in early pregnancy were at increased risk of developing GDM. A secondary aim was to characterize risk factors associated with the development of MeS. 

## 2. Subjects and Methods 

The study is a part of a cluster-randomized controlled trial, NELLI (counseling and lifestyle during pregnancy, ISRCTN33885819) [21]. A detailed description of the design and methods has been published previously [22]. The primary aim of the trial was to prevent GDM among pregnant women who were assessed in early pregnancy to have an increased risk of GDM. The study was conducted in primary health care maternity clinics in Western Finland in 2007–2009. The intervention included structured individual counselling on weight gain, diet, and physical activity by public health nurses during five routine visits to maternity clinics. The women in the control clinics received the usual maternal care, including some lifestyle advice.

Pregnant women were recruited by nurses at their first visit (8–12 weeks' gestation) in maternity clinics. Women were eligible if they had at least one of the following GDM risk factors: age ≥ 40 years, prepregnancy body mass index (BMI) ≥ 25 kg/m², GDM or any sign of glucose intolerance, a macrosomic baby (≥4500 g) in any previous pregnancy, or diabetes in first- or second-degree relatives. The main exclusion criteria were age < 18 years, a GDM diagnosis at 8–12 weeks' gestation, twin pregnancy, physical restrictions that precluded exercise, or a clinical history of chronic disease. A diagnosis of GDM was based on a two-hour 75-gram oral glucose tolerance test (OGTT) whose results met at least one of the following criteria: a fasting plasma glucose of ≥5.3 mmol/L, >10.0 mmol/L at one hour, or >8.6 mmol/L at two hours [14].

**Figure 1:**
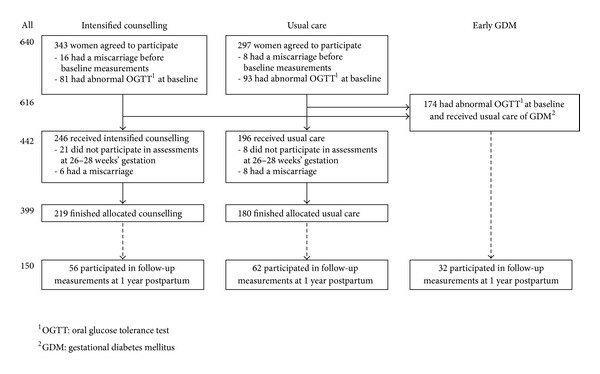
Flow diagram of the study, ending in followup assessments at one year postpartum. The two intervention groups (intensified counselling and usual care) and those with GDM diagnosed at early pregnancy (8–12 weeks' gestation) were invited for followup measurements at one year postpartum.

Six hundred forty pregnant women participated in baseline assessment at 8–12 weeks' gestation (Figure 1). Of them, 442 (69%) were eligible for the randomized clinical trial (RCT; intensified counselling or usual care), while 198 (31%) were excluded, most of them (*n* = 174, 88%) due to a GDM diagnosis at 8–12 weeks' gestation. At postpartum followup, MeS component data were available for 150 women. The intensified counselling, the usual care, and the early GDM (originally excluded from the RCT) groups were merged for present analysis.

Information on maternal measurements was obtained from the standard maternity cards. Height was measured at the first maternity care visit, and weight was measured at each maternity care visit and one year postpartum. Waist circumference was measured (the average of three measurements) at one year postpartum. Blood pressure was measured in duplicate at each maternity care visit and one year postpartum. Because 15% of the weight data from the first visit were missing, self-reported prepregnancy weight was used as the baseline weight. 

Blood specimens were taken for glucose, cholesterol, HDL cholesterol, and triglyceride analysis after a 12-hour fast, and a two-hour OGTT was performed at 8–12 and 26–28 weeks' gestation and one year postpartum. All blood analysis was performed at the UKK Institute. For glucose and lipid analysis, venous blood was drawn into citric acid/fluoride and EDTA tubes. During the OGTT, blood samples were taken between 60 and 120 min after the participants had drunk 75 g glucose in 330 mL water (Glucodyn, Ultimed, Finland). Plasma glucose concentrations were measured fresh within 24 hours after the OGTT, but plasma samples for lipid analysis were stored frozen at −80°C until analysed. Glucose, total cholesterol, HDL cholesterol, and triglyceride concentrations were determined in enzymatic assays using a Roche Cobas Mira Plus analyser. All testing was performed in duplicate. MeS was diagnosed according to the International Diabetes Federation (IDF) [6] and the National Cholesterol Education Program Adult Treatment Panel (NCEP ATP-III) [23] criteria. At one year postpartum, oral glucose tolerance was evaluated according to the American Diabetes Association (ADA) [24] and the World Health Organization (WHO) [25] criteria. The primary outcome of this study was the prevalence of MeS and its components at one year postpartum.

The background characteristics and descriptive information on components of metabolic syndrome are reported here as means and standard deviations (SDs) or frequencies and proportions. A multivariate logistic regression model was used to obtain odds ratios (ORs) and their 95% confidence intervals (95% CIs) to study associations between metabolic syndrome and its explanatory variables. Explanatory variables included were age (continuous), group (intensified counselling, usual care, and early GDM); and five GDM risk factors (as used in entrance criteria to the study, that is, BMI ≥ 25 kg/m², age ≥ 40 years, GDM or any sign of glucose intolerance in any previous pregnancy, a macrosomic baby [≥4500 g] in any previous pregnancy, and diabetes in first- or second-degree relatives). The results were considered to be statistically significant if *P* < 0.05. All analysis was performed with SPSS software (version 20.0).

## 3. Results

### 3.1. Background Characteristics

Before pregnancy, self-reported weight was 74.2 kg (range 50.0–120.0 kg), which was 0.9 kg less than the weight measured at the first maternal clinical visit at 8–12 weeks' gestation (75.1 kg, *n* = 127). The mean prepregnancy BMI was 26.7 kg/m² (range 18.1–39.5 kg/m²). At followup measurement; one year after delivery, the mean weight increase was 1.4 kg (range from −16.1 to 18.0 kg) compared with prepregnancy weight.  Table 1 shows that one year after delivery, the mean age of the women was 33.1 years (range 20–49 years) and the mean number of deliveries was 2.0 (range 1–8). The most common inclusion criteria for the study were prepregnancy overweight (66%) and diabetes in relatives (53%). Twenty-one percent (*n* = 30) smoked frequently or occasionally before pregnancy. 

### 3.2. Metabolic Syndrome and Its Components at One-Year Postpartum

The prevalence of MeS and its components one year after delivery in all women and in the intensified counselling, usual care, and abnormal OGTT groups is presented in Table 2. Three out of four women exceeded the waist circumference limit of 80 cm, and about half reached the limit of 88 cm. Compared to the intensified counselling group, there tended to be more abdominally obese (waist circumference ≥ 88 cm) women in the early GDM and usual care group. More than one-fourth of all and half of the women with early GDM had elevated fasting plasma glucose (**≥**5.6 mmol/L) at one year postpartum. One-fifth of all and one-fourth of women with early GDM also had elevated blood pressure. HDL cholesterol was reduced (≤1.3 mmol/L) among more than one-fourth of all women. 

On the other hand, almost one-third (31%) according to NCEP criteria and one-sixth (16%) according to IDF criteria did not meet the criteria for any MeS components. Most women (52% according to IDF and 63% according to NCEP) had one or two MeS components, while 8% (according to both IDF and NCEP) had four or five components. According to IDF criteria, the prevalence of MeS among the participants was 18% (*n* = 27) and according to NCEP criteria 16% (*n* = 24). In OGTT, at followup, 8 women (5%) had impaired glucose tolerance according to both WHO and ADA criteria. Seven had MeS according to both IDF and NCEP criteria. 

In multivariate logistic regression analysis, the risk of MeS in the group with early GDM tended to be higher compared to the intensified counselling group (OR3.4, 95% CI 1.0–11.3, *P* = 0.051) (Table 3). When analysed by trial inclusion criteria (GDM risk factors at baseline), prepregnancy overweight (BMI > 25 kg/m^2^) was a strong predictor for developing MeS (OR 3.0, 95% CI 1.0–9.2, *P* = 0.053).

### 3.3. Dropout Analyses

Measurements of MeS components at one year postpartum were available for 24% of the women who participated in the baseline measurements and for 32% of those who participated in the followup. Compared with the participants for whom the data for MeS diagnosis at followup were not available (*n* = 466), participants with data for MeS (*n* = 150) were more likely to belong to the usual care group (55% versus 45%, *P* = 0.032) and were less likely to be frequent smokers before pregnancy (10% versus 22%, *P* = 0.013). There were also some more women with GDM diagnosed at 26–28 weeks' gestation (abnormal OGTT) among women with MeS data available at one year postpartum than among those without MeS data (18% versus 10%, *P* = 0.064), but there were no differences in the occurrence of GDM at 8–12 weeks' gestation (20% versus 24% had early GDM, *P* = 0.393). Neither were there differences between these two groups in any other background characteristic (weight or BMI, age, parity, or education) or laboratory analysis other than OGTT at 26–28 weeks' gestation. 

## 4. Discussion

At one year postpartum, MeS was diagnosed in nearly one-fifth of the women who had an increased risk of gestational diabetes at the beginning of their pregnancy. Especially; prepregnancy overweight was associated with a higher risk of developing MeS. In a prospective Finnish population study among nonpregnant women aged 30–33, the prevalence of MeS was 7%–14% according to the IDF definition and 4%–11% according to the NCEP definition [26]. The prevalence of MeS was more common in our study due to our risk-group approach. According to other studies, the prevalence of MeS among parous women aged 30–40 is no more than 10% depending on country, criteria, and time after delivery [18, 19, 27]. 

Our study is one of the first followup studies on the prevalence of MeS after delivery among young women with risk factors for GDM. According to earlier studies, a history of GDM is strongly associated with a higher prevalence of MeS [16, 20, 27]. The present study suggests that the risk is high, also among women with risk factors for GDM in early pregnancy, but does not necessarily lead to GDM. GDM and MeS share some risk factors, such as overweight and a genetic tendency towards impaired glucose metabolism. Still, the development of MeS after glucose disorders during pregnancy has not been given as much attention as the increased risk of type 2 diabetes after GDM. In any case, the cardiometabolic risk factors in women at increased risk of GDM should also be followed after delivery.

In 2007, the prevalence of obesity among women aged 25–34 years in Finland was 11.1% [28]; in our subjects, one year after delivery, it was 2.5 times higher at 27%. Half of the subjects were abdominally obese (waist circumference **≥**88 cm). Abdominal obesity is one of the most important independent factors in development of metabolic syndrome [29]. Although overweight among young adults is on the rise worldwide [30], there are few studies of the prevalence of MeS among young adults.

The NELLI study [21] is one of the largest randomized controlled trials about preventing the development of gestational diabetes. The NELLI trial showed that the lifestyle counselling was effective in controlling the proportion of large-for-gestational-age newborns and improving the women's diet and had a minor effect on gestational weight gain and decrease in physical activity [21, 31–33]. Since participation in followup measurements at one year postpartum was low (24% of the original cohort) and the total number of participants was modest, the results must be interpreted carefully. Because of low number of participants, some findings were only borderline statistically significant.

There are many possible reasons for the loss. Among followup participants, there were more women from the usual care group and fewer frequent smokers before pregnancy. Participants in followup also were more likely to have been diagnosed with GDM in midpregnancy (26–28 weeks) as compared with the women who did not participate in MeS testing at followup. Women with small children may have found it difficult to find time to come in for testing, especially as it included the two-hour OGTT. Some subjects who showed up for followup testing may have been more health conscious, which is advocated by the fact that there was a smaller proportion of smokers among them. Some may have been concerned about ill health due to their GDM diagnosis during pregnancy. Nevertheless, some women with a GDM diagnosis were already being monitored by the healthcare system, and they may have refused to participate in our testing for that reason. Another reason for refusal was a new pregnancy, but the number of these women was unclear. This study was limited to Finnish women, and the results can only be extrapolated to Caucasian populations.

One limitation of this study is that, at the followup, the subjects were not queried about hormonal contraception or medications for hypertension or dyslipidemia, which influence the components of MeS. However, when medications were queried in the baseline, none of the women reported taking any cardiovascular medication. 

We used self-reported weight before pregnancy, which is not as reliable a measurement as using a scale. When self-reported weight was compared to the available weight measurement at the first maternity clinical visit, the mean difference was less than 1 kg, which could easily be explained by a minimal early pregnancy weight gain. 

## 5. Conclusion

Our study suggests that MeS at one year postpartum seems to occur more often among women who in early pregnancy had an increased risk of GDM. The most important factor associated with MeS seemed was prepregnancy overweight. This study suggests that especially women with an increased risk of GDM should be followed up on for cardiometabolic risk factors after delivery. Weight management or reduction before pregnancy and prevention of excessive weight gain during pregnancy are important for the prevention of GDM and of MeS. Overweight and obesity among pregnant women may increase, as the average maternal age is rising along with the obesity epidemic, and this represents an even greater challenge in following up on and managing risk factors for chronic diseases. There is a need for larger population studies on the prevalence of MeS among young women, especially among those who are at an elevated risk of GDM.

## Figures and Tables

**Table 1:** Background characteristics of women at one year postpartum. Means and standard deviations or frequencies (and proportions) of participants (*n* = 150).

Age (years)	33.1 ± 4.9
<30	37 (25)
30–34	60 (40)
≥35	53 (35)
Weight (kg)	75.6 ± 15.3
BMI^a^ (kg/m^2^)	27.2 ± 5.0
BMI ≥ 25 kg/m^2^	88 (64)
BMI ≥ 30 kg/m^2^	37 (27)
Education	
Basic or secondary education	48 (32)
Polytechnic education	60 (40)
University degree	41 (28)
Parity	2.0 ± 1.2
1	56 (37)
2-3	82 (55)
≥4	12 (8)
GDM^b^ risk criteria (at 8–12 weeks' pregnancy, *n* = 148)	
BMI^a^ ≥ 25 kg/m^2^	97 (66)
Macrosomic child in any previous pregnancy	8 (5)
GDM^b^in any previous pregnancy	25 (17)
Diabetes in first- or second-degree relatives	78 (53)
Age ≥ 40 years	6 (4)

^a^BMI: body mass index.

^b^GDM: gestational diabetes mellitus.

**Table 2:** Components of metabolic syndrome (MeS) and prevalence of MeS by two criteria in all women and in the intensified counselling, usual care, and abnormal OGTT groups at one year postpartum. Means and standard deviations or frequencies (and proportions) of participants.

	All (*n* = 135–150)	Intensified counselling (*n* = 49–56)	Usual care (*n* = 56–62)	Early GDM^a^ (*n* = 30–32)
Waist circumference (cm)	88.8 ± 11.4	86.7 ± 11.5	88.8 ± 10.2	92.7 ± 12.7
Waist ≥ 80 cm	102 (74)	36 (69)	43 (77)	23 (77)
Waist ≥ 88 cm	69 (50)	20 (39)	30 (54)	19 (63)
Fasting glucose (mmol/L)	5.4 ± 0.4	5.3 ± 0.4	5.3 ± 0.4	5.7 ± 0.5
Fasting glucose ≥ 5.6 mmol/L	43 (29)	11 (20)	16 (26)	16 (50)
Systolic pressure (mmHg)	116 ± 11	114 ± 9	116 ± 12	118 ± 12
Diastolic pressure (mmHg)	74 ± 9	72 ± 7	76 ± 10	75 ± 10
Blood pressure ≥ 130 or ≥85 mmHg	23 (17)	4 (8)	12 (21)	7 (23)
HDL cholesterol (mmol/L)	1.50 ± 0.34	1.43 ± 0.30	1.57 ± 0.37	1.49 ± 0.30
HDL cholesterol ≤ 1.3 mmol/L	40 (27)	17 (32)	14 (23)	9 (28)
Triglycerides (mmol/L)	0.97 ± 0.43	0.93 ± 0.37	0.94 ± 0.44	1.06 ± 0.49
Triglycerides ≥ 1.7 mmol/L	12 (8)	3 (6)	4 (7)	5 (16)
Metabolic syndrome (IDF)^b^	27 (18)	6 (11)	11 (18)	10 (31)
Metabolic syndrome (NCEP)^c^	24 (16)	4 (7)	10 (16)	10 (31)

^a^GDM: gestational diabetes mellitus.

^b^International Diabetes Federation.

^c^National Cholesterol Education Adult Treatment Panel III.

**Table 3:** Occurrence of metabolic syndrome by its explanatory variables (group, age, and five GDM risk factors). Odds ratios (ORs), 95% confidence intervals (CIs), and *P* values, *n* = 150. Multivariate logistic regression model.

	OR (95% CI)	*P* value
Group (reference = the intensified counselling)		
Usual care	1.5 (0.5 to 4.6)	0.48
Early GDM^a^	3.36 (1.00 to 11.4)	0.051
Age (continuous)	1.0 (0.9 to 1.2)	0.44
BMI^b^ ≥ 25 kg/m^2^ (prepregnancy)	3.0 (1.0 to 9.2)	0.053
A macrosomic baby (≥4500 g) in any previous pregnancy	0.9 (0.1 to 6.5)	0.91
GDM^a^ or any sign of glucose intolerance in any previous pregnancy	2.6 (0.9 to 7.6)	0.077
Type 1 or 2 diabetes in first- or second-degree relatives	1.6 (0.6 to 4.00)	0.32
Age ≥ 40 years	0.9 (0.1 to 11.8)	0.92

^a^GDM: gestational diabetes.

^b^BMI: body mass index.
